# Differences in Use of a Patient Portal Across Sociodemographic Groups: Observational Study of the NHS App in England

**DOI:** 10.2196/56320

**Published:** 2024-11-13

**Authors:** Sukriti KC, Chrysanthi Papoutsi, Claire Reidy, Bernard Gudgin, John Powell, Azeem Majeed, Felix Greaves, Anthony A Laverty

**Affiliations:** 1 Imperial College London School of Public Health London United Kingdom; 2 University of Oxford Nuffield Department of Primary Care Health Sciences Oxford United Kingdom; 3 Public Member Cambridge United Kingdom

**Keywords:** digital health, patient portals, technological health divide, eHealth, inequality, observational, ecological, England, app, patient portal, disparities, deprivation, demographics, long-term health care, negative binomial regression model, intervention, patient support, general practice, digital technology, patient, youth

## Abstract

**Background:**

The adoption of patient portals, such as the National Health Service (NHS) App in England, may improve patient engagement in health care. However, concerns remain regarding differences across sociodemographic groups in the uptake and use of various patient portal features, which have not been fully explored. Understanding the use of various functions across diverse populations is essential to ensure any benefits are equally distributed across the population.

**Objective:**

This study aims to explore differences in the use of NHS App features across age, sex, deprivation, ethnicity, long-term health care needs, and general practice (GP) size categories.

**Methods:**

We used weekly NHS App use data from the NHS App dashboard for 6386 GPs in England from March 2020 to June 2022. Negative binomial regression models explored variations in weekly rates of NHS App features used (registrations, log-ins, prescriptions ordered, medical record views, and appointments booked). Outcomes were measured as weekly rates per 1000 GP-registered patients, and we conducted separate models for each outcome. Regression models included all covariates mentioned above and produced incident rate ratios, which we present here as relative percentages for ease of interpretation. GP-level covariate data on sociodemographic variables were used as categorical variables in 5 groups for deprivation (Q1=least deprived practices and Q5=most deprived practices) and 4 groups for all other variables (Q1=least deprived practices and Q4=most deprived practices).

**Results:**

We found variations in the use of different features overall and across sociodemographic categories. Fully adjusted regression models found lower use of features overall in more deprived practices (eg, Q5 vs Q1: registrations=–34%, log-ins=–34.9%, appointments booked=–39.7%, medical record views=–32.3%, and prescriptions ordered=–9.9%; *P*<.001). Practices with greater proportions of male patients also had lower levels of NHS App use (eg, Q4 vs Q1: registration=–7.1%, log-in=–10.4%, and appointments booked=–36.4%; *P*<.001). Larger practices had an overall higher use of some NHS App features (eg, Q4 vs Q1: registration=3.2%, log-ins=11.7%, appointments booked=73.4%, medical record views=23.9%, and prescriptions ordered=20.7%; *P*<.001), as well as those with greater proportions of White patients (eg, Q4 vs Q1: registration=1.9%, log-ins=9.1%, appointments booked=14.1%, medical record views=28.7%, and prescriptions ordered=130.4%; *P*<.001). Use patterns varied for practices with greater proportions of patients with long-term health care needs (eg, Q4 vs Q1: registrations=–3.6%, appointments booked=–20%, and medical record views=6%; *P*≤.001).

**Conclusions:**

This study highlights that the use of the NHS App features varied across sociodemographic groups. In particular, it is used less by people living in more deprived areas. Tailored interventions and patient support are required to ensure that any benefits from the NHS App are spread equally throughout the population.

## Introduction

Digital technologies are rapidly gaining global momentum as drivers for transformative changes in health care [1,2]. In England, strategies to modernize the National Health Service (NHS) in part involved the introduction of “digital-first” options for all patients and key digitalization milestones such as the implementation of comprehensive electronic health care records across all NHS trusts by 2025 [3,4]. As part of these initiatives, the NHS App was introduced in 2019 as a digital route to a range of primary care services for all general practice (GP)–registered patients in England aged 13 years and older [5,6]. It is a patient portal that offers patients access to their health care data and facilitates interaction with their care providers using functions that allow viewing GP records, requesting repeat prescriptions, managing GP appointments, checking symptoms, registering for organ donation, and setting data sharing preferences [6]. Subsequently, additional features have also been introduced that enable accessing vaccination records, receiving messages regarding care, managing secondary care appointments, and viewing hospital waiting times [7]. Prior to 2019, portal use varied by country and was growing in England, albeit without one consistent national portal. From 2015 onward, patient portals had to be offered by GPs, and there were different iterations of national schemes offering services such as appointment booking.

Research on electronic health care record access using patient portals underscores their potential to improve patient satisfaction, enable self-management, and support empowerment by actively engaging patients in their health care decisions [8-10]. There are also reported benefits in several aspects of care, such as disease awareness, medication adherence, and patient safety [8,11]; however, their impacts on health inequalities are less well understood [12,13]. These not only relate to patient portal adoption, which is generally lower in older people [14,15], ethnic minority groups [14,16], and those from a lower socioeconomic status [15], but they are also reflected in sustained engagement trends and the use of different portal functionalities [16]. Research highlights that users may show preferences for specific patient portal features, and their ability to engage with certain functions is influenced by factors such as age, digital literacy, health conditions, and personal health care needs [17-19]. For example, a study looking at patient portal adoption by older adults revealed a notable interest among this group in using functions enabling access to health information and appointment booking. However, there were also reported issues surrounding the usability of the technology and a need for increased user support [20]. In a separate study, younger adults and female individuals exhibited greater use of patient portal features overall. However, older patients used functions associated with education more frequently and individuals experiencing higher poverty engaged with features related to billing and insurance more [19]. Such variations in patient engagement with different patient portal features may indicate an inherent user preference and the influence of the perceived value of the technology based on patient-specific needs.

Some existing research has further explored these issues. Analyses of data from 2015 to 2017 reported that patients with multiple health conditions placed a higher value on patient access portals than other patients [21]. Research focused on appointment booking and medication ordering using patient surveys from 2018 to 2020 has found these services are more likely to be used by patients with long-term health conditions and higher socioeconomic status [22]. Previous analyses by our group that used data until May 2021 focused on the impacts of COVID-19 events, but we also found higher NHS App registration rates among larger-sized, less deprived, and less ethnically diverse GP practices and younger people [23]. This study, however, assessed differences in NHS App registration only and no other app functions.

Such differences across sociodemographic groups in patient engagement need careful consideration as digital innovations may follow an inverse care law, where the availability and use of health care are inversely related to the population who need it most [24,25]. Furthermore, as national platforms, such as the NHS App, continue to evolve and play more prominent roles in health care delivery [4], prioritizing sustained patient engagement and participation beyond initial uptake is essential to achieve meaningful success. Therefore, identifying patterns of use of the various app features and understanding how different population groups are engaging with those functions is important. This study thus aims to explore the patterns of uptake of various NHS App functions and analyze differences in use across different sociodemographic groups.

## Methods

### Data Sources

We conducted an ecological study using data at the GP practice level exploring differences in the use of NHS App features by the GP-registered population in England. The outcome variables were weekly registrations, log-ins, prescriptions ordered, medical record views, and appointments booked. NHS App use data were available from March 2020 to June 2022, provided by the NHS Digital team as anonymized daily logs from the NHS App dashboard [26].

Data on the sociodemographic characteristics of the GP-registered population and their health care needs at the GP level were used as covariates. Data on the age and sex of all GP-registered populations were obtained from the NHS Digital website, which is a reliable source for the collection and processing of national data across the health and social care sector in the United Kingdom [27]. We excluded data for those younger than 15 years of age, as the NHS App is not offered to younger age groups [6], and the resultant total for all GP-registered male and female patients was combined to calculate the GP practice size. Population aged 15-34 years was used as the age identifier for analyses, and the percentage of male patients was used as the identifier for sex.

Data on long-term health care needs were extracted from the GP Patient Survey, which is a survey of approximately 2.4 million adult GP-registered patients in the United Kingdom [28]. Population-weighted positive responses to the question “Do you have any long-term physical or mental health conditions, disabilities, or illnesses?” obtained from the GP Patient Survey database were included.

Information on ethnic composition and the Index of Multiple Deprivation (IMD) quintile were obtained from the Fingertips public health profile, which is a web-based repository of a range of health and social care indicators mapped as public health profiles at local and national levels [29]. The identifier for ethnicity included the percentage of the population who reported their ethnicity as “White.”

Data obtained from these public health data sources were then ranked into quartiles (4 groups). Quartile 1 (Q1) included practices with the lowest population percentage for the given variable, and quartile 4 (Q4) was the highest. The only exception to this was the IMD split, which followed the Office of National Statistics method of ranking into quintiles (5 groups) [30], where quintile 1 (Q1) included practices with the lowest IMD score (ie, least deprived practices), and quintile 5 (Q5) included practices with the highest (ie, most deprived practices).

For analyses, covariate data were linked to the NHS App data using the GP practice code, which serves as a unique identifier code for each GP practice in England. Practices with incomplete data on practice size or those with missing area codes were removed to avoid errors during analysis (n=126). Practices with fewer than 200 registered patients were also excluded (n=20) to remove practices that service atypical populations such as specific sexual health services or services for homeless people. A comparison of characteristics of included and excluded practices (Table S1 in Multimedia Appendix 1) found that excluded practices had greater proportions of male patients and younger patients, and had substantially lower mean practice sizes. The population covered by our data was similar to the general population in terms of age, sex, and ethnicity (Table S2 in Multimedia Appendix 1). The ranges of categorical variables used for analyses are shown in Table S3 in Multimedia Appendix 1. The percentage of male patients ranged from 28.57% to 48.99% in the lowest quartile 1 compared with from 51.05% to 94.56% in quartile 4. The percentage of White patients ranged from 9.50% to 75.93% in the lowest quartile compared with from 97.30% to 99.56% in quartile 4. The number of registered patients in quartile 1 ranged from 255 to 5394 compared with from 12,033 to 110,443 in quartile 4.

### Analyses

We had complete weekly use data for a period between March 23, 2020, and June 27, 2022, for a total of 6386 GP practices in England. Outcomes were measured as weekly rates per 1000 registered GP-registered patients and as cumulative totals at the end of the study period. Negative binomial regression models using incident rate ratios were used to explore subgroup differences in the patterns of NHS App use by patient sociodemographic characteristics, their long-term health care needs, and GP practice size. The regression models compared variations in app use for the different covariates in comparison to the reference group Q1 (ie, for IMD=least deprived practices, and for all other covariates=practices with the lowest population percentage for the given variable). Incident rate ratio values were then used to calculate the percentage change to report the relative difference between the different covariate quintiles. We also present analyses modeling covariates linearly. For these, we still use IMD in 5 categories, but the percentage of the practice population that is male, the percentage in the youngest age group, the percentage of White ethnicity, and the percentage with a long-term health illness or disability were all modeled as linear variables. Practice size coefficients are presented per 1000 increase. All statistical analyses were performed using Stata 17.0 (StataCorp LLC).

### Ethical Considerations

Ethical approval was provided by the Imperial College London Research Ethics Committee (reference 21IC7292).

## Results

### Overview

We present cumulative totals for all NHS App features in Table 1.

**Table 1:** Cumulative total National Health Service (NHS) App functions used between March 23, 2020, and June 27, 2022.

NHS App feature	Cumulative total use, n	Rate per 1000 patients, mean (SD)
Registrations	24,168,835	3.27 (3.96)
Log-ins	447,976,852	59.30 (65.26)
Appointments booked	1,757,175	0.21 (0.80)
Medical record views	117,962,559	15.24 (15.83)
Prescriptions ordered	21,324,472	2.80 (3.61)
Patient population (week of June 20-27, 2022)	61,099,213	—^a^

^a^Not applicable.

### Registrations

Across the whole study period, there were 24,168,835 NHS App registrations (Table 1).

**Figure 1:**
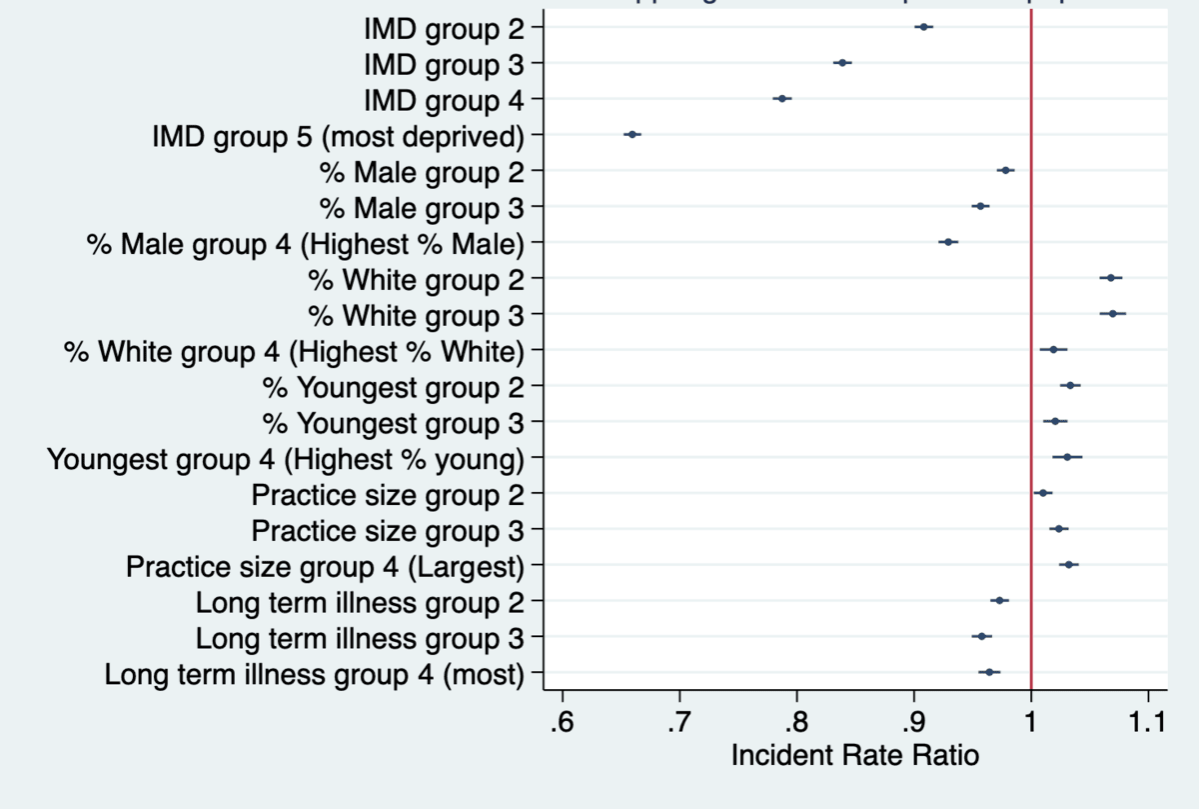
Weekly NHS App registration rates per 1000 GP-registered patients for the different covariate quantiles. IMD Q1 (reference group)=least deprived practice and IMD Q5=most deprived practices. For all other covariates, Q1=practices with the lowest population percentage for the given variable and Q4=practices with the highest population percentage for the given variable. GP: general practice; IMD: Index of Multiple Deprivation; NHS: National Health Service.

Results for all outcomes from fully adjusted models are shown in Table S2 in Multimedia Appendix 1 and results for NHS App registrations are presented in Figure 1. These show strong associations between IMD and app registration, with lower rates of registration in all quintiles than in Q1 (ie, least deprived; test for trend *P*<.001). For example, registration rates were 34% lower in Q5 than in Q1 (*P*<.001). Registrations were also lower in practices with higher proportions of male patients (eg, 7.1% lower in Q4 than in Q1; *P*<.001) and in practices with greater proportions of patients with long-term health needs (eg, 3.6% lower in Q4 compared with Q1, *P*<.001; test for trend *P*<.001).

Registrations were higher in practices with higher proportions of White patients (eg, 1.9%, lower in Q4 compared with Q1; *P*<.001) and those with more patients from the youngest age group (aged 15-34 years; eg, 3.1%, lower in Q4 compared with Q1; *P*<.001). Registration rates were also higher in larger-sized practices (eg, 3.2% lower in Q4 than in Q1, *P*<.001; test for trend *P*<.001).

### Log-ins

**Figure 2:**
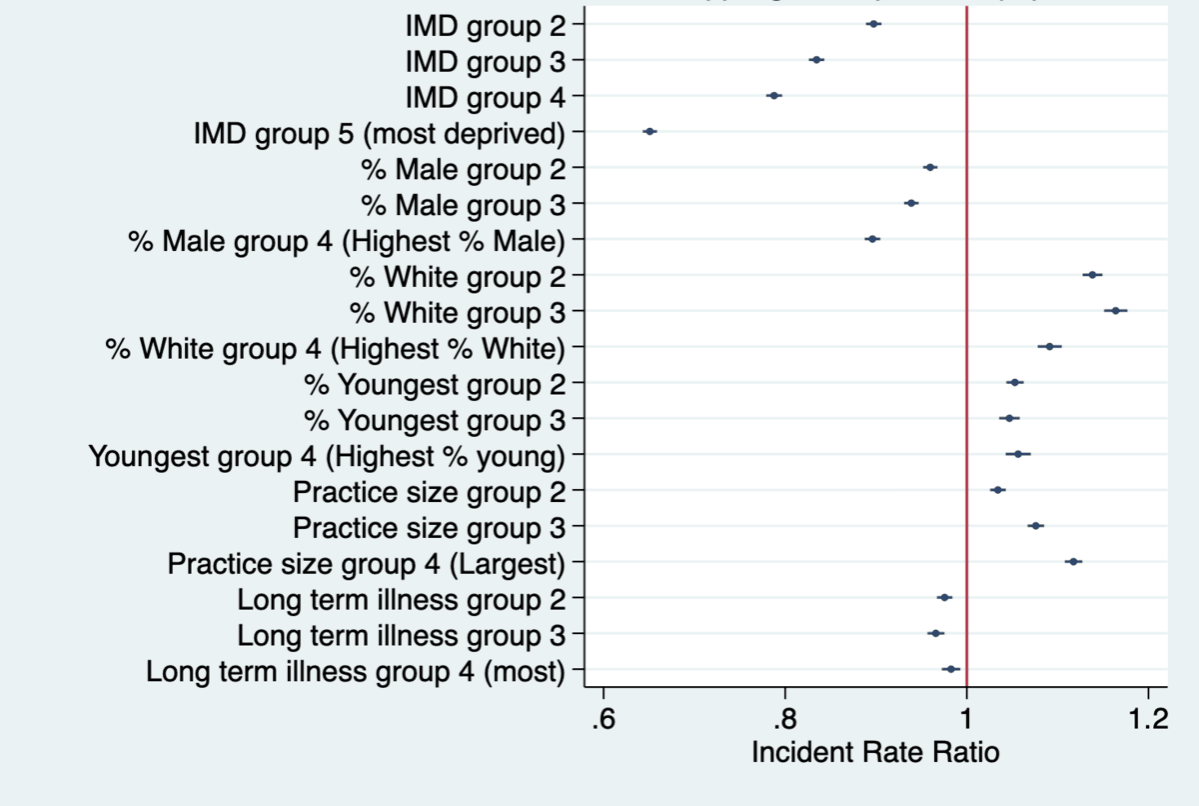
Weekly NHS App log-in rates per 1000 GP-registered patients for the different covariate quantiles. IMD Q1 (reference group)=least deprived practice and IMD Q5=most deprived practices. For all other covariates, Q1=practices with the lowest population percentage for the given variable and Q4=practices with the highest population percentage for the given variable. GP: general practice; IMD: Index of Multiple Deprivation; NHS: National Health Service.

Across the whole study period, there were 447,976,852 NHS App log-ins (Table 1). Results showing subgroup differences in NHS App registrations using the fully adjusted models are presented in Figure 2 and they show strong associations between IMD and app log-ins, with lower log-in rates in all quintiles compared to Q1 (ie, least deprived; test for trend *P*<.001). For example, log-in rates were 34.9% lower in Q5 than in Q1 (*P*<.001). Log-ins were also lower in practices with higher proportions of male patients (eg, 10.4% lower in Q4 than in Q1; *P*<.001) and in practices with greater proportions of patients with long-term health needs (eg, 3.4% lower in Q3 than in Q1 and 1.7% lower in Q4 than in Q1, *P*=.001; test for trend *P*<.001).

App log-ins were generally higher in practices with a higher proportion of White patients (eg, 16.4% higher in Q2 than in Q1 and 9.1% higher in Q4 than in Q1; *P*<.001) and in those with more patients from the youngest age group (eg, 5.6% higher in Q4 than in Q1; *P*<.001). NHS App log-in rates were also higher in the larger-sized practices (eg, 11.7% higher in Q4 than in Q1, *P*<.001; test for trend *P*<.001).

### Appointments Booked

**Figure 3:**
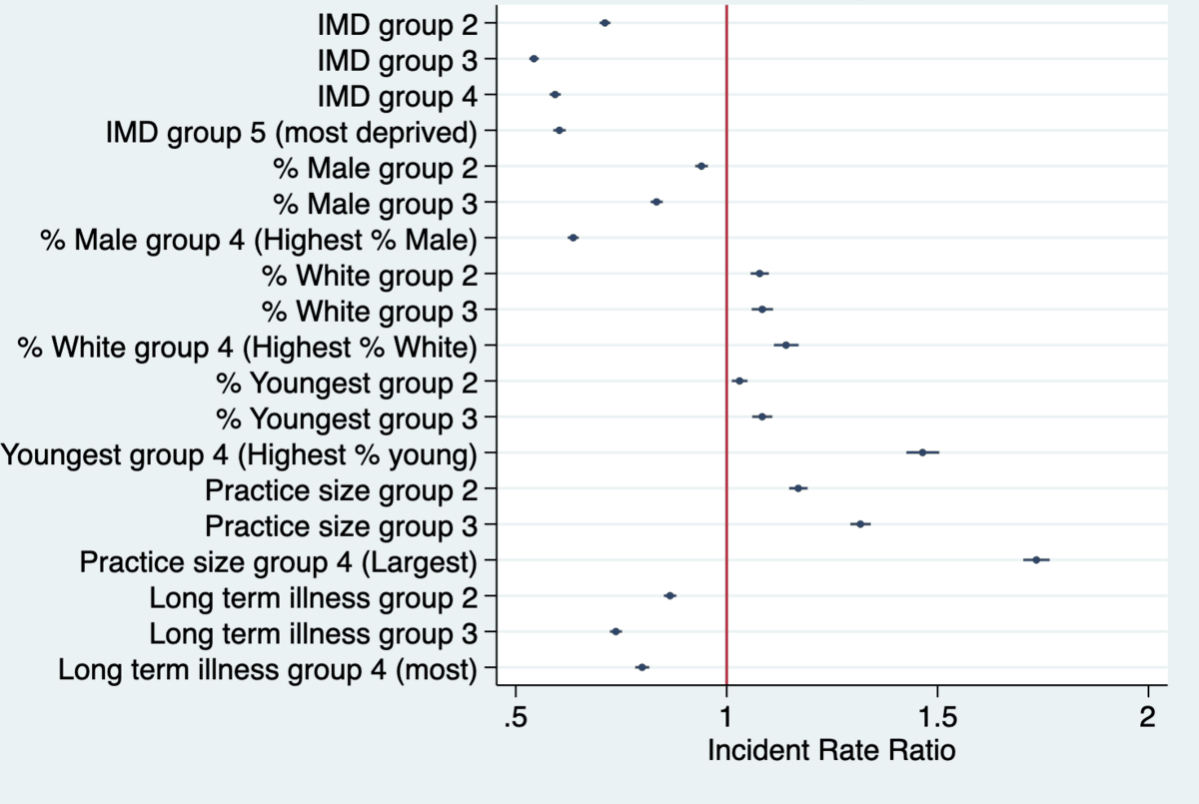
Weekly rates of appointments booked using the NHS App per 1000 GP-registered patients for the different covariate quantiles. IMD Q1 (reference group)=least deprived practice and IMD Q5=most deprived practices. For all other covariates, Q1=practices with the lowest population percentage for the given variable and Q4=practices with the highest population percentage for the given variable. GP: general practice; IMD: Index of Multiple Deprivation; NHS: National Health Service.

Across the whole study period, there were 1,757,975 GP appointments booked using the NHS App. Results showing subgroup differences in the rates of appointment booked using the fully adjusted models are presented in Figure 3 and they show strong associations between IMD and appointments booked, with lower rates of appointment booking rate in all quintiles than in Q1 (ie, least deprived; test for trend *P*<.001). For example, rates of appointments booked were 45.7% lower in Q2 than in Q1 and 39.7% lower in Q5 compared to Q1 (*P*<.001). Appointment booking rates were also lower in practices with higher proportions of male patients (eg, 36.4% lower in Q4 than in Q1; *P*<.001) and in those with more patients with long-term health needs (eg, 20% lower in Q4 than in Q1; *P*=.001; test for trend *P*<.001).

The rates of appointments booked were higher in practices with the highest proportion of White patients (eg, 14.1% higher in Q4 than Q1, *P*<.001) and those with the most patients from the youngest age group (ie, 46.5% higher in Q4 than Q1; *P*<.001). The rates of appointments booked were also higher among the larger-sized practices (eg, 73.4% in Q4 than in Q1, *P*<.001; test for trend *P*<.001).

### Medical Record Views

**Figure 4:**
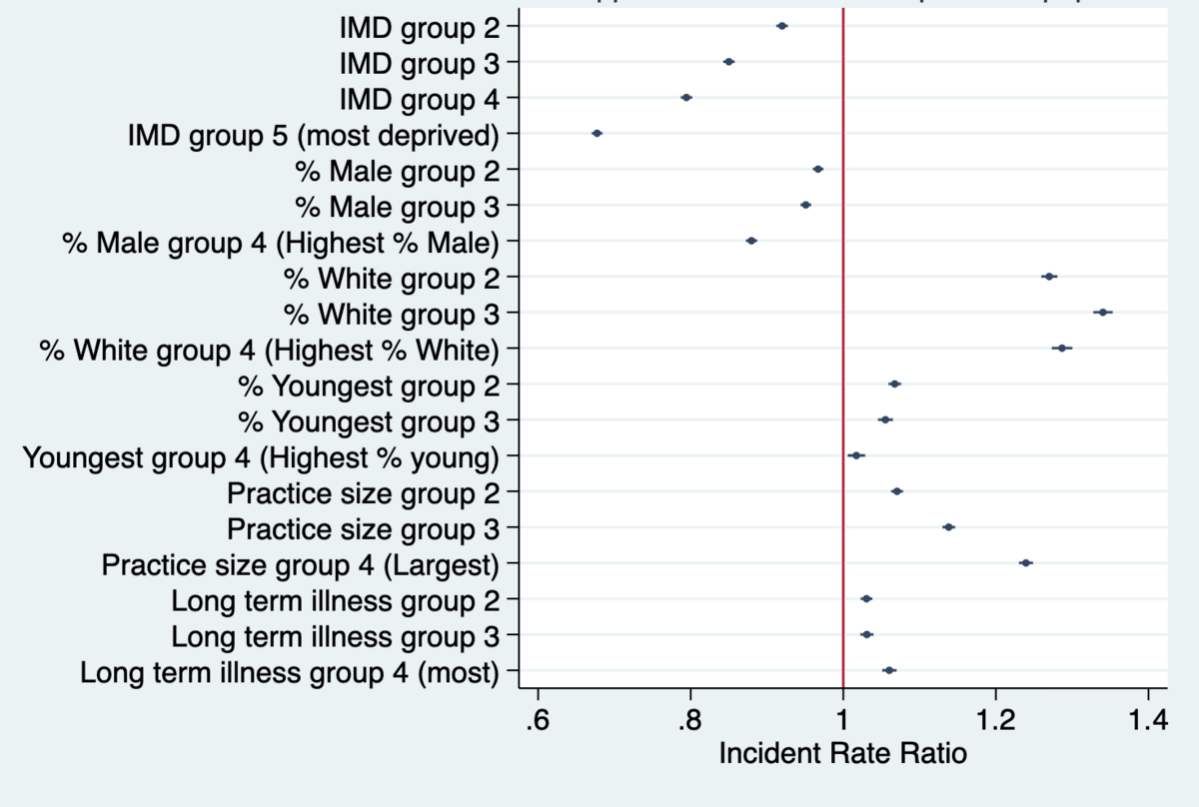
Weekly rates of medical record views using the NHS App per 1000 GP-registered patients for the different covariate quantiles. IMD Q1 (reference group)=least deprived practice and IMD Q5=most deprived practices. For all other covariates, Q1=practices with the lowest population percentage for the given variable and Q4=practices with the highest population percentage for the given variable. GP: general practice; IMD: Index of Multiple Deprivation; NHS: National Health Service.

Across the whole study period, there were 117,962,559 medical record views through the NHS App. Results showing subgroup differences in the rates of medical records viewed using the fully adjusted models are presented in Figure 4 and they show strong associations between IMD and medical record views, with higher rates in all quintiles than in Q1 (ie, least deprived; test for trend *P*<.001). For example, rates of medical record views were 32.3% lower in Q5 than in Q1 (*P*<.001). They were also lower in practices with higher proportions of male patients (eg, 12% lower in Q4 than in Q1, *P*<.001; test for trend *P*<.001).

Rates of medical record views were higher in practices with a higher proportion of White patients (eg, 34% higher in Q2 than in Q1 and 28.7% higher in Q4 than in Q1; *P*<.001) and in those with more patients from the youngest age group (eg, 6.7% higher in Q2 than in Q1, *P*<.001; and 1.7% higher in Q4 than in Q1, *P*=.003). The rates of medical records views were also higher in larger-sized practices (eg, 23.9% higher in Q4 than in Q1, *P*<.001) and in practices with more people with long-term health care needs (eg, 6% in Q4 than in Q1, *P*<.001; test for trend *P*<.001).

### Prescriptions Ordered

**Figure 5:**
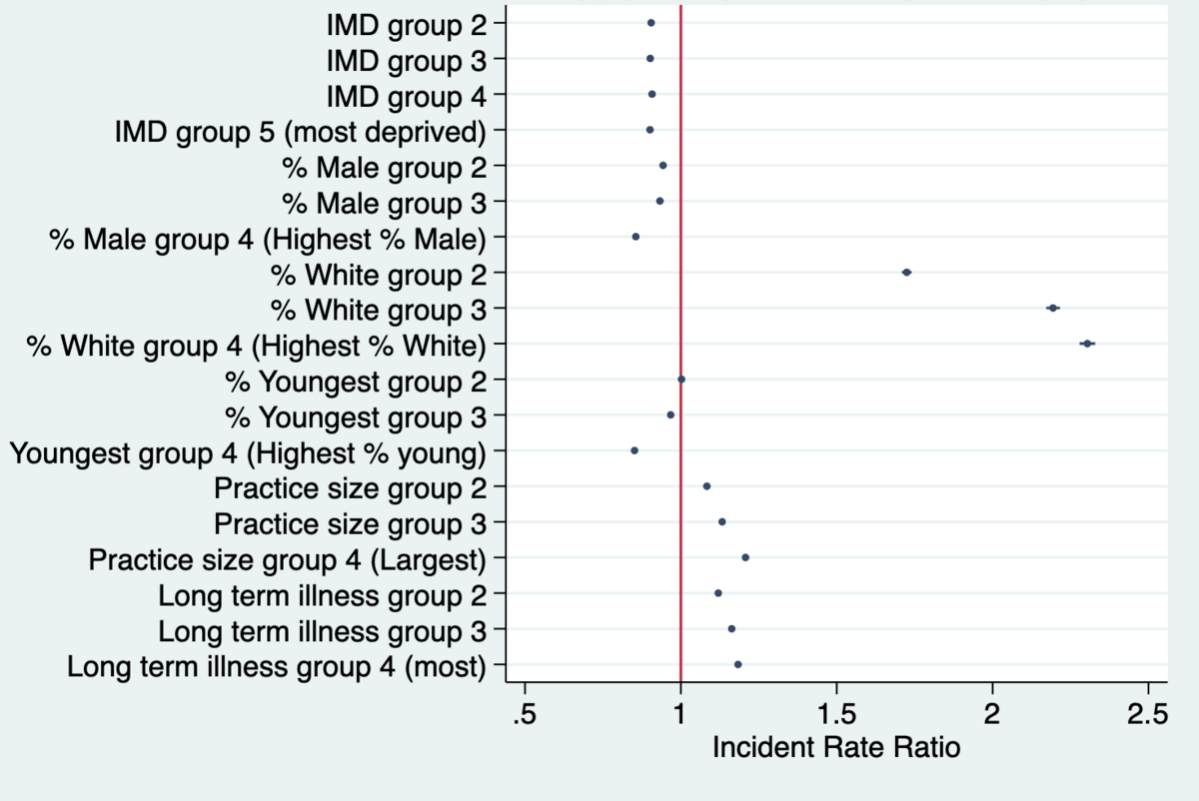
Weekly rates of prescriptions ordered using the NHS App per 1000 GP-registered patients for the different covariate quantiles. IMD Q1 (reference group)=least deprived practice and IMD Q5=most deprived practices. For all other covariates, Q1=practices with the lowest population percentage for the given variable and Q4=practices with the highest population percentage for the given variable. GP: general practice; IMD: Index of Multiple Deprivation; NHS: National Health Service.

Across the whole study period, there were 21,324,472 prescriptions ordered using the NHS App. Results showing subgroup differences in the rates of prescriptions ordered using the fully adjusted models are presented in Figure 5 and they show strong associations between IMD and rates of prescriptions ordered, with lower rates in all quintiles compared to Q1 (ie, least deprived; test for trend *P*<.001). For example, rates of prescriptions ordered were 9.9% lower in Q5 than in Q1 (*P*<.001). Prescription order rates were also lower in practices with higher proportions of male patients (eg, 14. 5% lower in Q4 than in Q1; *P*<.001) and in practices with a higher proportion of people aged 15-34 years (eg, 14.8% lower in Q4 than Q1; *P*<.001).

The rates of prescriptions ordered were significantly higher in practices with a higher proportion of White patients (eg, 119.4% higher in Q3 than in Q1 and 130.40% higher in Q4 than in Q1; *P*<.001) and in larger practice sizes (eg, 20.7% higher in Q4 than in Q1, *P*<.001). The rates of prescriptions ordered were also higher in practices with more people with long-term health care needs (eg, 18.3% higher in Q4 than in Q1, *P*<.001; test for trend *P*<.001).

### Additional Analyses

Additional analyses using covariates except for IMD as linear variables found similar patterns (Table S4 in Multimedia Appendix 1). These found each one percent increase in the percentage of male patients was associated with lower use of all outcomes (eg, –2.78%; *P*<.001 for prescription ordering). Each 1% increase in the percentage of patients in the youngest age group was associated with a –0.45% (*P*<.001) decrease in prescription ordering but higher levels of other app functions. Increased percentage of patients of White ethnicity and increased practice sizes were associated with greater use of all app functions. Increased percentage of patients with a long-term illness was associated with fewer registrations (–0.28%; *P*<.001), fewer log-ins (–0.28%; *P*<.001), and appointment booking (–0.77%; *P*<.001), but higher rates of medical record views (0.01; *P*<.001) and prescription ordering (0.61; *P*<.001).

We also conducted analyses controlling for season which gave similar results for the sociodemographic factors of interest (Table S5 in Multimedia Appendix 1).

## Discussion

### Principal Findings

This study explored the NHS App and found there to be high levels of patient engagement, although this differed across sociodemographic categories. The most prominent finding was the clear deprivation gradient with lower rates of use of the various NHS App features among practices in more deprived areas. In contrast, the use of the different features was higher among the largest-sized practices and in those with greater proportions of White patients. There was a 130% difference in the rates of prescriptions ordered among practices with the highest proportion of White patients compared to the lowest. In practices with greater proportions of younger patients, appointment booking rates were higher, but rates of prescription ordered were lower. Similarly, in practices with greater proportions of patients with long-term health care needs, rates of medical record views and prescriptions ordered were higher, but the rates of appointment booking, registrations, and log-ins were generally lower.

Some other results also stand out as particularly striking; for example, with ordering prescriptions digitally—one of the most commonly used features—there is a strong ethnicity gradient, with practices with a higher proportion of White patients using this function more than twice as much as practices with a lower proportion of White patients.

### Strengths and Limitations

This is the first study to explore variations in the use of different NHS App features in relation to different sociodemographic groups using data over 27 months. It provides a comprehensive overview of public uptake of the NHS App using a nationally representative sample of the GP-registered population in England and offers a crucial understanding of digital health engagement in a real-world setting. While these findings suggest an unequal pattern of patient engagement with the NHS App features in relation to patient age, sex, ethnicity, deprivation, and patient health care needs, there are limitations in using population-level data to understand individual-level differences. While the NHS App allows patients to select their data sharing preferences, individual-level data are not yet available for research, which restricts a more direct evaluation of these use patterns [6].

There are challenges in capturing and controlling the effects of the influences of wider confounding factors, such as regulatory changes, competing availabilities of similar commercial digital health platforms, and changes in strategic decisions [31], which have implications for our research findings. We included several sociodemographic variables and their proxies identified in consultation with the wider research team and the Public and Patient Involvement group, but the full range of indicators categories (eg, additional age ranges and ethnic groups) and broader socioeconomic variables were excluded due to a lack of available data. Specifically, we were unable to use data on patients on prescribed medication at each GP practice, and rates of prescriptions ordered use the whole population as a denominator. While some variables are encompassed within existing indicators (eg, income and education are parts of IMD value calculation), these factors may operate in silos to influence how people engage with digital resources [1]. Furthermore, exclusions were applied to remove practices with incomplete data, as well as removing practices serving unconventional populations. This along with the ecological nature of the analysis may affect the generalizability of results and present missed opportunities to capture the demography these practices serve and their distinct challenges.

### Comparison With Prior Work on NHS App Use

The results of this study extend the findings of our previous research [23] and indicate differences in patient engagement with the NHS App across sociodemographic groups. NHS Digital highlights that the NHS App currently has over 30 million registrations, with over 65 million patient record views and 22 million repeat prescription orders through the app in 2022 [32]. While our data support these findings by highlighting high use across all app features, evidence of a varied pattern of app function use among diverse population groups is congruent with wider research in the field [21,22]. Most notably, reduced use of the different functions in more deprived and ethnically diverse practices reinforces concerns related to the digital inverse care law [25]. A previous study exploring differences in the uptake of NHS primary care services, including the NHS App, highlighted reduced use of both the NHS App and web-based patient portals among the 4.27 million patients living in the most deprived quintiles in England [33]. As the NHS plans for the app to be a primary way to access services, reduced use among deprived groups risks widening health disparities—that is, avoidable differences in outcomes between groups.

Although the diffusion of innovation theory suggests that some of these differences may decrease as the technology is more widely used by the general population [34], certain patient portal features may continue to have differential adoption based on individual capacity and need [19]. Discrepancies in the use of the various functions, as highlighted by our results, such as younger adults showing increased appointment bookings but decreased prescription requests, underscore these complexities. Younger adults generally have a better capacity for engagement with digital health technology, yet their overall health care use tends to be lower, primarily because their health care needs are generally less pronounced [35]. Therefore, the reduction in prescription orders through the NHS App among this group may reflect a good health status overall. The evidence of increased uptake of certain transactional services, such as appointment booking, could be indicative of their technological literacy and it may have been influenced by their motivation to use these services. While we could not measure these associations directly for the NHS App, they warrant further exploration in future studies.

In congruence with previous studies, our results also highlight notable differences in different app feature use among individuals with long-term health conditions [21,22]. While appointment booking rates were lower among this group, medical record views and prescription orders were higher, signaling heightened patient engagement with features catering to their ongoing health care needs [36,37]. The lower levels of appointment booking could be associated with the configuration of this feature, which is contingent upon the specific provisions implemented by individual practices. It could also be linked to variations in patient self-care ability as studies highlight that individuals with long-term health care needs often depend on their social networks and caregivers for support and they could potentially benefit from delegated digital access for care coordination [12,38]. Although the NHS App offers access to linked profiles for family members and carers, the availability of these proxy access entitlements relies on individual GP practice provisions and this could be examined in future research [39].

Nevertheless, our results show variation in the use of the app overall and specific functions by various patient characteristics, suggesting opportunities for design, policy, and practice change. This might include targeted marketing of the app and its functions toward specific groups, as well as targeted outreach and education for groups such as older people, ethnic minorities, and those with lower incomes. This could include, for example, digital literacy programs to equip people from these groups with the skills needed to use the NHS App more effectively. Other measures to reduce differences in the use of NHS App could include helplines for those who need support or advice; and collaboration with community groups that work with underserved populations. Furthermore, if those with better capacity, interest, and opportunity to engage with technology use digital services more, it may reduce pressures on the health care system and offer cost-saving opportunities to improve care for those who continue to benefit from in-person support [40]. This may be particularly relevant to a patient-facing tool, such as the NHS App, which is available nationally, offering a range of functions for diverse population groups. Therefore, identifying users and nonusers of the NHS App and supporting engagement with features most relevant to their specific needs may be crucial to creating equal outcomes for all [41] and needs further research. There is also a need for assessment of the uptake trends of the NHS App contingent on continued policy efforts and facilitation strategies to understand its relevance, user satisfaction, and equitable access, contributing to the overall success of such digital health initiatives.

### Conclusions

We found ongoing high levels of patient engagement with the various NHS App functions, although these differed across sociodemographic groups. NHS App uptake and use had a strong deprivation gradient as well as influences of patient age, ethnicity, and health care needs, indicating the requirement for dedicated efforts to meaningfully engage different population groups. Further research is also needed to explore how these differences affect health outcomes.

This study contributes to the growing body of evidence on digital health engagement and lays the groundwork for future research and policy making. It underscores the importance of continuous monitoring, evaluation, and adaptation of digital health initiatives to ensure they meet the evolving needs of diverse populations and contribute to equitable health outcomes for all. The NHS App has the potential to be a valuable tool for patient care in the NHS in England, but it is important to ensure that all individuals have the opportunity to use the app effectively.

## Appendix

Multimedia Appendix 1 Supplementary tables.

## Abbreviations

GP: general practice
IMD: Index of Multiple Deprivation
NHS: National Health Service
