# Supplementary material for: Differences in Use of a Patient Portal Across Sociodemographic Groups: Observational Study of the NHS App in England
Source: J Med Internet Res. 2024 Nov 13;26:e56320. doi: 10.2196/56320 (PMC11602757; doi:10.2196/56320)
Supplement: Multimedia Appendix 1 [file jmir_v26i1e56320_app1.docx]

|  | Included | Excluded | p for difference |
| --- | --- | --- | --- |
| **% in most deprived quintile** | 3.2 | 2.8 | 0.434 |
| **% Male** | 50.4 | 56.2 | <0.001 |
| **% youngest** | 25.6 | 27.5 | 0.034 |
| **% White** | 83.3 | 79.9 | 0.037 |
| **% Long term health** | 35.4 | 36.9 | 0.022 |
| **Practice size (mean)** | 9567 | 5437 | <0.001 |

**Table S1: Comparison of GP practices included in analyses and those excluded**

**Table S2: Comparison of included population to general population (from ONS statistics)**

|  | In sample | In England population |
| --- | --- | --- |
| % Male | 49.82 | 49.00 |
| % aged 15-34 | 25.58 | 25.25 |
| % aged 35 - 54 | 27.25 | 26.26 |
| % aged 55 - 74 | 21.96 | 22.50 |
| % aged 75+ | 8.45 | 8.62 |
| % White | 83.26 | 82.00 |

**Table S3: Ranges for categorical variables**

| **Characteristic** | **Range** |
| --- | --- |
| % male quartile 1 (lowest % male patients) | 28.57 to 48.99 |
| % male quartile 2 | 49.00 to 49.81 |
| % male quartile 3 | 49.81 to 51.09 |
| % male quartile 2 (highest % male patients) | 51.05 to 94.56 |
| % White quartile 1(lowest % white patients) | 9.50 to 75.93 |
| % White quartile 2 | 75.97 to 92.83 |
| % White quartile 3 | 92.84 to 97.03 |
| % White quartile 1(highest % white patients) | 97.30 to 99.56 |
| Practice size quartile 1 (smallest) | 255 to 5394 |
| Practice size quartile 2 | 5396 to 8280 |
| Practice size quartile 3 | 8288 to 12030 |
| Practice size quartile 4 (largest) | 12033 to 110443 |
| % long term illness quartile 1 (lowest) | 1 to 30 |
| % long term illness quartile 2 | 31 to 36 |
| % long term illness quartile 3 | 37 to 41 |
| % long term illness quartile 4 (highest) | 42 to 68 |

**Table S4: Results from negative binomial models of NHS App use using linear models (separate models for each outcome)**

|  | **Registrations** | | **Logins** | | **Appointment booking** | |
| --- | --- | --- | --- | --- | --- | --- |
|  | **% change** | **p value** | **% change** | **p value** | **% change** | **p value** |
| **IMD least deprived quintile** | ref | ref | ref | ref | ref | ref |
| **Quintile 2** | -9.99 | <0.001 | -11.04 | <0.001 | -31.69 | <0.001 |
| **Quintile 3** | -16.96 | <0.001 | -17.07 | <0.001 | -48.65 | <0.001 |
| **Quintile 4** | -21.63 | <0.001 | -20.75 | <0.001 | -43.59 | <0.001 |
| **IMD most deprived quintile** | -34.58 | <0.001 | -34.84 | <0.001 | -42.74 | <0.001 |
| **% Male** | -1.46 | <0.001 | -2.01 | <0.001 | -5.94 | <0.001 |
| **% youngest** | 0.61 | <0.001 | 0.88 | <0.001 | 1.35 | <0.001 |
| **% White** | 0.30 | <0.001 | 0.61 | <0.001 | 0.07 | 0.005 |
| **Practice size (1000 increase)** | 0.06 | 0.004 | 0.49 | <0.001 | 2.50 | <0.001 |
| **% Long term illness** | -0.28 | <0.001 | -0.28 | <0.001 | -0.77 | <0.001 |
|  | **Medical record views** | | **Prescription ordering** | |  |  |
|  | **% change** | **p value** | **% change** | **p value** |  |  |
| **IMD least deprived quintile** | ref | ref | ref | ref |  |  |
| **Quintile 2** | -8.42 | <0.001 | -9.05 | <0.001 |  |  |
| **Quintile 3** | -15.02 | <0.001 | -9.81 | <0.001 |  |  |
| **Quintile 4** | -19.43 | <0.001 | -7.54 | <0.001 |  |  |
| **IMD most deprived quintile** | -32.07 | <0.001 | -10.39 | <0.001 |  |  |
| **% Male** | -2.45 | <0.001 | -2.78 | <0.001 |  |  |
| **% youngest** | 0.51 | <0.001 | -0.46 | <0.001 |  |  |
| **% White** | 0.86 | <0.001 | 2.01 | <0.001 |  |  |
| **Practice size (1000 increase)** | 1.14 | <0.001 | 1.04 | <0.001 |  |  |
| **% Long term illness** | 0.10 | <0.001 | 0.61 | <0.001 |  |  |

Results from negative binomial regressions with covariates modelled linearly for a 1 percent increase. Percentage in youngest age group based on patients aged 16-24. Mean practice size presented per 1000 patient increase.

**Table S5: Results from negative binomial models of NHS App use using linear models (separate models for each outcome) controlling for season**

|  | **Registration rate** | | **Login rate** | | **Appointment booking rate** | | **Medical record rate** | | **Prescription ordering rate** | |  |
| --- | --- | --- | --- | --- | --- | --- | --- | --- | --- | --- | --- |
| **Winter** | ref | ref | ref | ref | ref | ref | ref | ref | ref | ref | |
| **Spring** | 118.90 | <0.001 | -25.91 | <0.001 | -56.39 | <0.001 | 24.23 | <0.001 | -60.90 | <0.001 | |
| **Summer** | 93.86 | <0.001 | 8.79 | <0.001 | -6.01 | <0.001 | -4.69 | <0.001 | -38.21 | <0.001 | |
| **Autumn** | 73.23 | <0.001 | 59.50 | <0.001 | -0.56 | 0.504 | 31.83 | <0.001 | -15.61 | <0.001 | |
| **% male quartile 1 (lowest % male patients)** | ref | ref | ref | ref | ref | ref | ref | ref | ref | ref | |
| **% male quartile 2** | -8.77 | <0.001 | -10.60 | <0.001 | -29.49 | <0.001 | -7.99 | <0.001 | -9.99 | <0.001 | |
| **% male quartile 3** | -15.57 | <0.001 | -17.09 | <0.001 | -46.49 | <0.001 | -14.99 | <0.001 | -10.14 | <0.001 | |
| **% male quartile 2 (highest % male patients)** | -20.61 | <0.001 | -21.87 | <0.001 | -40.90 | <0.001 | -20.49 | <0.001 | -9.28 | <0.001 | |
| **% White quartile 1(lowest % white patients)** | -33.27 | <0.001 | -36.02 | <0.001 | -40.67 | <0.001 | -32.46 | <0.001 | -10.23 | <0.001 | |
| **% White quartile 2** | ref | ref | ref | ref | ref | ref | ref | ref | ref | ref | |
| **% White quartile 3** | -2.18 | <0.001 | -4.15 | <0.001 | -5.51 | <0.001 | -3.38 | <0.001 | -6.31 | <0.001 | |
| **% White quartile 1(highest % white patients)** | -4.23 | <0.001 | -6.18 | <0.001 | -16.60 | <0.001 | -4.98 | <0.001 | -7.00 | <0.001 | |
| **% White quartile 1(lowest % white patients)** | -6.77 | <0.001 | -10.59 | <0.001 | -36.66 | <0.001 | -12.07 | <0.001 | -14.61 | <0.001 | |
| **% youngest quartile 2** | ref | ref | ref | ref | ref | ref | ref | ref | ref | ref | |
| **% youngest quartile 3** | 5.90 | <0.001 | 13.68 | <0.001 | 7.99 | <0.001 | 26.81 | <0.001 | 72.34 | <0.001 | |
| **% White quartile 1(highest % white patients)** | 5.97 | <0.001 | 16.31 | <0.001 | 8.44 | <0.001 | 34.02 | <0.001 | 121.75 | <0.001 | |
| **Practice size quartile 1 (smallest)** | 1.29 | 0.036 | 8.61 | <0.001 | 14.22 | <0.001 | 28.43 | <0.001 | 131.96 | <0.001 | |
| **Practice size quartile 2** | ref | ref | ref | ref | ref | ref | ref | ref | ref | ref | |
| **Practice size quartile 3** | 3.18 | <0.001 | 5.27 | <0.001 | 1.94 | 0.047 | 6.82 | <0.001 | 0.01 | 0.984 | |
| **Practice size quartile 4 (largest)** | 2.09 | <0.001 | 4.82 | <0.001 | 7.61 | <0.001 | 5.65 | <0.001 | -3.02 | <0.001 | |
| **% long term illness quartile 1 (lowest)** | 3.51 | <0.001 | 5.53 | <0.001 | 46.05 | <0.001 | 1.95 | 0.001 | -14.06 | <0.001 | |
| **% long term illness quartile 2** | ref | ref | ref | ref | ref | ref | ref | ref | ref | ref | |
| **% long term illness quartile 3** | 0.97 | 0.021 | 3.49 | <0.001 | 17.18 | <0.001 | 7.08 | <0.001 | 8.39 | <0.001 | |
| **% long term illness quartile 4 (highest)** | 2.27 | <0.001 | 7.93 | <0.001 | 33.57 | <0.001 | 13.94 | <0.001 | 13.44 | <0.001 | |
| **% male quartile 1 (lowest % male patients)** | 3.33 | <0.001 | 12.08 | <0.001 | 76.64 | <0.001 | 24.07 | <0.001 | 21.77 | <0.001 | |
| **% male quartile 2** | ref | ref | ref | ref | ref | ref | ref | ref | ref | ref | |
| **% male quartile 3** | -2.64 | <0.001 | -2.66 | <0.001 | -13.39 | <0.001 | 2.97 | <0.001 | 11.46 | <0.001 | |
| **% male quartile 2 (highest % male patients)** | -4.20 | <0.001 | -3.74 | <0.001 | -26.21 | <0.001 | 2.99 | <0.001 | 15.83 | <0.001 | |
| **% White quartile 1(lowest % white patients)** | -3.54 | <0.001 | -2.01 | <0.001 | -20.16 | <0.001 | 5.96 | <0.001 | 17.59 | <0.001 | |
